# Effects of the dietary nonfiber carbohydrate content on lactation performance, rumen fermentation, and nitrogen utilization in mid-lactation dairy cows receiving corn stover

**DOI:** 10.1186/s40104-018-0239-z

**Published:** 2018-03-14

**Authors:** Zihai Wei, Baoxin Zhang, Jianxin Liu

**Affiliations:** Institute of Dairy Science, MoE Key Laboratory of Molecular Animal Nutrition, College of Animal Sciences, Zhejiang University, Hangzhou, 310058 People’s Republic of China

**Keywords:** Corn stover, Dairy cows, Lactation performance, Nonfiber carbohydrate, Rumen fermentation

## Abstract

**Background:**

Corn stover (CS) is an abundant source of feed for livestock in China. However, it is low in nutritional value that we have been seeking technologies to improve. Previous studies show that non-fiber carbohydrate (NFC) might limit the utilization of a CS diet by lactating dairy cows. Thus, this study was conducted to investigate the lactation performance and rumen fermentation characteristics in lactating cows consuming CS with two contents of NFC compared to an alfalfa hay-containing diet. Twelve Holstein cows were used in a replicated 3 × 3 Latin square design with three dietary treatments: (1) low-NFC diet (NFC = 35.6%, L-NFC), (2) high-NFC diet (NFC = 40.1%, H-NFC), and (3) alfalfa hay diet (NFC = 38.9%, AH).

**Results:**

Intake of DM was lower for cows fed H-NFC compared to L-NFC and AH, while the milk yield was higher in AH than in H-NFC and L-NFC (*P* < 0.01). The feed efficiency (milk yield/DM intake, 1.15 vs. 1.08, *P* < 0.01) were greater for cows fed H-NFC than L-NFC. The contents of milk protein and lactose were not different among the groups (*P* > 0.11), but milk fat content was higher for cows fed H-NFC and L-NFC compared to AH (*P* < 0.01). The rumen ammonia nitrogen concentration and the concentrations of urea nitrogen in blood and milk were lower for cows fed H-NFC and AH compared to L-NFC (*P* < 0.05). The concentrations of rumen propionate and total volatile fatty acids were different among groups (*P* < 0.05) with higher concentration for cows fed AH compared to H-NFC and L-NFC, and acetate concentration tended to be different among groups (*P* = 0.06).

**Conclusions:**

From the results obtained in this study, it was inferred that the increased NFC content in a diet containing corn stover can improve the feed efficiency and benefit the nitrogen conversion.

## Background

With the rapid development of the Chinese dairy industry with its population of 14 million [1], the requirement for high quality forage increased greatly with nearly 1 million tons of alfalfa hay imported from abroad recently [2]. In contrast, approximately 220 million tons of corn stover (CS) were produced annually [3], indicating a great potential forage source for dairy cows. Compared to high quality forage, the deficiency in available energy and low content of crude protein (CP) can reduce the degradability of low quality forage [4]. The cows fed an alfalfa diet produced greater milk and milk protein compared to cows fed a CS diet [5, 6]. There is a need to improve the utilization of low quality forage in feeding strategies.

Non-fiber carbohydrates (NFC), including starch, sugars and pectin, are estimated by subtracting CP, neutral detergent fiber (NDF), and ether extract (EE) from organic matter [7]. Increasing levels of dietary NFC or starch can improve lactation performance and is beneficial for utilization of energy and nitrogen (N) in dairy cows [8, 9]. Supplementation with an NFC source (e.g. corn starch) in a CS diet provided readily fermentable carbohydrates in the rumen and achieved a similar microbial protein (MCP) yield and metabolizable protein (MP) supply compared to an alfalfa diet [10, 11]. In a study by Wang et al. [6], higher milk protein yield in the alfalfa-fed cows compared to CS-fed cows was partly attributed to higher NFC content in the AH diet.

Therefore, it was hypothesized that NFC content may be an important factor that limits utilization of a CS diet by lactating dairy cows. The objective of this study was to investigate the differences in lactation performance, blood metabolites, and rumen fermentation in dairy cows consuming CS with low or high NFC content compared to an alfalfa-containing diet.

## Methods

### Animals, feeds and design

The experimental procedures were approved by the Animal Care Committee at Zhejiang University (Hangzhou, China). Twelve Holstein cows in mid-lactation (159 ± 15 d in milk; 704 ± 72 kg of BW; mean ± SD) were used in a replicated 3 × 3 Latin square design with three dietary treatments: (1) low-NFC diet (NFC = 35.6%, L-NFC), (2) high-NFC diet (NFC = 40.1%, H-NFC), and (3) alfalfa hay diet (NFC = 38.9%, AH) (Table 1). In both L-NFC and H-NFC diets, corn stover was included at approximately 15% of total dietary dry matter (DM) to replace alfalfa hay in the AH diet. All the experimental diets were isonitrogenous and isocaloric, and they were formulated according to the NRC [7] to meet the requirements of cows for milk production of 29 kg/d with 3.9% milk fat and 3.3% milk protein.

**Table 1:** Ingredients and chemical compositions of the 3 experimental diets

Items	Treatment^a^		
	AH	L-NFC	H-NFC
Dietary ingredient, g/kg of DM			
Alfalfa hay	149	0.0	0.0
Oat hay	49.5	44.1	46.8
Corn stover	0.0	147	148
Corn silage	267	257	266
Ground corn grain	297	229	299
Wheat bran	49.5	103	5.4
Soybean meal	99.0	177	175
Cottonseed meal	49.5	4.9	21.2
Premix^b^	39.6	38.2	39.4
Chemical composition^c^, % of DM			
Dry matter	46.2	46.4	45.5
Crude protein	15.0	14.9	14.5
RDP, % of Crude protein	64.2	63.3	62.1
RUP, % of Crude protein	35.8	36.7	37.9
Ether extracts	3.8	3.6	3.6
Crude ash	7.7	8.3	7.5
Neutral detergent fiber	34.6	37.5	34.3
Acid detergent fiber	19.5	19.9	19.0
Non-fiber carbohydrate^c^	38.9	35.6	40.1
NE_L_, MJ/kg	6.73	6.57	6.69
Diet price^d^, $/kg DM	0.388	0.353	0.360

^a^AH = Total mixed rations containing alfalfa hay. L-NFC = Total mixed rations containing corn stover with low content of non-fiber carbohydrates; H-NFC = Total mixed rations containing corn stover with high content of non-fiber carbohydrates similar to AH

^b^Contained (per kilogram of DM): 250,000 IU of vitamin A, 50,000 IU of vitamin D, 1,100 IU of vitamin E, 600 mg of Fe, 650 mg of Cu, 3,000 mg of Zn, 630 mg of Mn, 17 mg of Se, 36 mg of I, 8 mg of Co, 150-180 g of salt, water ≤100 g

^c^Non-fiber carbohydrate = 100 – % neutral detergent fiber − % crude protein − % ether extract − % ash; RDP, RUP and NE_L_ were estimated according to CPM dairy 3.0

^d^Diet price was calculated based on the ingredient purchase prices by the farm

All cows were housed in a tie-stall barn and had free access to drinking water. The corn silage we used was from the same silo, and all the other ingredients used in the experiment were from the same batch which were prepared at the beginning of the experiment, thus the diets were consistent from day to day through the experiment period. Total mixed rations (TMR) were offered ad libitum to allow at about 10% orts. The cows were fed 3 times daily at 07:00, 13:30 and 19:30 h, and milked 3 times daily at 08:00, 14:30 and 20:30 h, respectively. Each period lasted for 21-d each with the first 14 d dedicated to adaptation. To change the diet between periods, cows were gradually changed to new diet in four days, with 25% daily increase to the new diet. Milk yield and milk composition were recorded during d 15–21, and rumen fluid samples were taken on d 19 of each period.

### Sampling, measurements, and analyses

The amounts of feed offered and refused by each cow were weighed and recorded daily during d 15–21 of each period. The samples of TMR and orts were taken three times at each feeding time on d 18 of each period. The collected samples were stored in sealed plastic bags at − 20 °C before analysis. The samples were dried at 65 °C for 48 h and ground in a Wiley mill with a 2-mm mesh screen (Thomas-Wiley Laboratory Mill) followed by 1 mm mesh screen (Tecator 1093, Hoganas, Sweden). After grinding, the samples of TMR and orts were composited by period, treatment and cow, respectively, and then used for chemical analysis. Corn stover and alfalfa hay were sampled at the beginning and end of each period, and the samples were stored and ground using the same approach as TMR samples. All the samples were analyzed for DM (105 °C for 5 h), CP (#988.05), ether extract (EE, #920.39), crude ash (#942.05), and acid detergent fiber (ADF, #973.18) according to AOAC methods [12]. The NDF content was analyzed using the methods of Van Soest et al. [13], with sodium sulfite and amylase added. An ANKOM^2000^ fiber analyzer (Ankom Technology Corp., Macedon, USA) was used to extract and filter NDF and ADF. Ingredients and chemical compositions of the diets are presented in Table 1, and the chemical composition of alfalfa hay and corn stover used in the study are presented in Table 2.

**Table 2:** Nutrient composition of alfalfa hay and corn stover used in the experimental diets

Items, % of DM	Alfalfa hay	Corn stover
Dry matter	90.0 ± 0.56	90.8 ± 0.51
Crude protein	15.1 ± 1.26	6.3 ± 0.36
Crude ash	8.4 ± 1.25	9.7 ± 0.04
Ether extracts	2.2 ± 0.62	1.1 ± 0.59
Neutral detergent fiber	52.1 ± 2.74	74.4 ± 3.39
Acid detergent fiber	37.0 ± 4.41	42.6 ± 1.62
Non-fiber carbohydrate^a^	22.2 ± 1.23	8.5 ± 3.67

^a^Non-fiber carbohydrate = 100 – % neutral detergent fiber − % crude protein − % ether extract − % ash

Milk yield was recorded daily during the sampling periods, and milk samples were collected on d 17, 18, and 19 of each period with milk-sampling devices (Waikato Milking Systems NZ Ltd., Hamilton, New Zealand). The samples of each day were composited proportionally (4:3:3 according to three times of milking). Samples were stored with added bronopol tablets (milk preservative; D & F Control System Inc., San Ramon, CA) for later analysis of milk protein, fat, lactose, total solids, urea N (MUN) and somatic cell counts using a Combi Foss FT+ instrument (Foss Electric, Hillerød, Denmark).

Diet cost in current study was calculated based on the ingredient purchase prices by the farm. The feed cost per kilogram of milk was also calculated by dividing the feed costs by milk yield.

Blood samples were obtained from the mammary vein into heparinized vacuum tubes every 6 h on d 18 and d 19 during each period, then centrifuged at 3,000×*g* for 15 min to obtain plasma, and stored at − 20 °C for later analysis. Plasma samples were analyzed using an Auto Analyzer 7020 instrument (Hitachi High-technologies Corporation, Tokyo, Japan) with colorimetric commercial kits (Ningbo Medical System Biotechnology Co., Ltd., Ningbo, China) to determine the blood concentrations of total protein, albumin, urea N (BUN), glucose, cholesterol, non-esterified fatty acid (NEFA) and β-hydroxybutyric acid (BHBA).

Rumen fluid samples (approximately 150 mL) were collected approximately 3 h after morning feeding using an oral stomach tube [14] on d 19 of each experimental period. The rumen fluid pH was measured immediately using a pH meter (FE20-FiveEasy Plus™; Mettler Toledo Instruments Co. Ltd., Shanghai, China). Triplicate 1-mL samples were acidified with 20 μL of orthophosphoric acid; and the other triplicate 1-mL samples were acidified with 6 *N* HCl and frozen at − 20 °C for later analysis of volatile fatty acids (VFA) and ammonia N (NH_3_-N), as described by Hu et al. [15].

### Estimation of microbial protein

Urinary purine derivatives (PD) were used to indirectly estimate the MCP yield in the rumen [16]. Spot urine samples from each cow were collected four times daily at approximately 06:00, 10:00, 14:00 and 18:00 h on d 17 of each experimental period. The daily urine samples were pooled by cow, and 20 mL of each subsample was acidified immediately with 80 mL of 0.072 *N* H_2_SO_4_ and stored at − 20 °C for later analysis. The PD (allantoin and uric acid) were analyzed using the procedures of Chen and Gomes [16] and creatinine was analyzed using a colorimetric picric acid assay [17]. Creatinine has been validated as a marker to estimate urine volume [18], and was assumed to be excreted at a rate of 29 mg/kg of BW for calculating the urine volume excretion rate [19]. Urea N was analyzed using a colorimetric method [20].

### Statistical analysis

Data on lactation performance, DM intake, feed cost per kilogram of milk, and feed efficiency were analyzed using the PROC MIXED procedure with repeated measurement in SAS software [21]. The model included the square, period, treatment, day, interaction of treatment by square, period and day as the fixed effects as well as cow within the square as a random effect. Plasma parameters were analyzed using the same procedure in SAS, except sampling hour instead of day was used as a repeated measure. The covariance structure was selected according to best fit with Schwarz’s Bayesian information criterion. For rumen fermentation variables and urine parameters, the model included square, period, treatment, and interaction of treatment by square and period as the fixed effects, and cow within the square as a random effect. The results listed as least squares means were separated using the pdiff option when the fixed effects were significant. A significant difference was declared as having *P* < 0.05, and 0.05 ≤ *P* ≤ 0.10 was defined as a statistical trend.

## Results

### Feed intake and lactation performance

The results of feed intake and lactation performance are presented in Table 3. The DM intake was lower (*P* < 0.01) for cows fed H-NFC compared to L-NFC and AH, with no difference between L-NFC and AH. Milk yield was higher (*P* < 0.01) for cows fed AH compared to H-NFC and L-NFC, although the difference was not significant (*P* = 0.20) in energy-corrected milk yield (ECM) among the three diets. Feed efficiency (milk yield/DM intake) was higher (*P* < 0.01) for cows fed H-NFC and AH compared to cows fed L-NFC; and the efficiency based on ECM (ECM/DM intake) was numerally higher for cows fed H-NFC than L-NFC, though the difference was not significant among three groups (*P* = 0.16). The contents of milk protein (*P* = 0.65) and lactose (*P* = 0.11) were not different among three groups, but milk fat content was higher (*P* < 0.01) in cows fed H-NFC and L-NFC compared to AH-fed cows, and the content of total solids was higher (*P* < 0.01) in cows fed L-NFC compared to cows fed AH with no difference between cows fed H-NFC and AH or L-NFC. The MUN was lower (*P* < 0.01) for cows fed H-NFC and AH compared to cows fed L-NFC. The feed cost per kg of milk was different among groups (*P <* 0.01) with lowest value for cows fed H-NFC (Table 3).

**Table 3:** Lactation performance in dairy cows fed 3 experimental diets

Items	Treatment^d^			SEM	*P*-value
	AH	L-NFC	H-NFC		
DM intake, kg/d	21.9^a^	21.5^a^	20.1^b^	0.44	< 0.01
Milk yield, kg/d	24.8^a^	23.2^b^	22.8^b^	0.76	< 0.01
ECM^e^, kg/d	27.7	27.0	26.4	1.01	0.20
Milk composition, %					
Fat	3.90^b^	4.25^a^	4.11^a^	0.12	< 0.01
Protein	3.48	3.52	3.50	0.06	0.56
Lactose	5.00	4.95	4.98	0.06	0.11
Total solid	12.6^b^	12.9^a^	12.8^ab^	0.17	< 0.01
Milk urea nitrogen, mg/dL	18.1^b^	20.3^a^	18.3^b^	0.72	< 0.01
Feed efficiency, kg/kg					
Milk/DM intake	1.15^a^	1.08^b^	1.15^a^	0.03	< 0.01
ECM/DM intake	1.28	1.27	1.32	0.04	0.16
Somatic cell count, ×10^3^/mL	94.8	64.2	70.6	20.6	0.26
Feed cost per kg milk, $/kg	0.342^a^	0.331^b^	0.319^c^	0.01	< 0.01

^a-c^Least squares means within a row with different superscripts differ significantly (*P* < 0.05)

^d^AH = Total mixed rations containing alfalfa hay. L-NFC = Total mixed rations containing corn stover with a low content of non-fiber carbohydrates; H-NFC = Total mixed rations containing corn stover formulated to have similar non-fiber carbohydrate concentrations as the AH diet

^e^ECM = Energy-corrected milk. ECM (kg) = 0.3246 × milk yield (kg) + 13.86 × fat yield (kg) + 7.04 × protein yield (kg) [39]

### Nitrogen utilization

Urinary PD, and estimated MCP yield were not different (*P* > 0.10) among 3 groups (Table 4), but the urinary urea nitrogen concentration tended to be lower in AH and H-NFC compared to L-NFC (*P* = 0.09), and the estimated urine volume of cows fed H-NFC was lower than cows fed L-NFC and AH (*P* < 0.05). The results for N utilization are presented in Table 5. Similar to the DM intake, N intake was lower (*P* < 0.01) for cows fed H-NFC compared to L-NFC and AH, with no difference between L-NFC and AH. The N excreted in the urine (g/d) was lower (*P* = 0.03) in the H-NFC-fed cows than that in cows fed L-NFC and AH; and the ratio of urinary N to N intake was relatively lower in cows fed H-NFC than that in cows fed L-NFC and AH (Table 5). There was no difference in the ratio of milk N (*P* = 0.20) among the three groups.

**Table 4:** Urinary nitrogen, purine derivatives (PD), and estimated microbial protein (MCP) supply to the dairy cows fed 3 experimental diets

Items^d^	Treatment^c^			SEM	*P*-value
	AH	L-NFC	H-NFC		
Urine volume, L/d	34.3^a^	32.7^a^	28.6^b^	1.52	0.02
Urinary PD, mmol/d					
Allantoin	358	377	325	22.0	0.26
Uric acid	60.8	59.2	52.7	4.97	0.22
Endogenous PD	52.6	52.7	52.5	1.28	0.68
MCP, g/d	1690	1766	1516	117	0.31
Urea nitrogen, mg/dL	670	730	664	30.1	0.09

^a-b^ Least squares means within a row with different superscripts differ significantly (*P* < 0.05)

^c^AH = Total mixed rations containing alfalfa hay; L-NFC = Total mixed rations containing corn stover with a low content of non-fiber carbohydrates; H-NFC = Total mixed rations containing corn stover formulated to have similar non-fiber carbohydrate concentrations as the AH diet

^d^Urine volume (L/d) = BW (kg) × 29 (mg/d)/creatinine (mg/L) [18]; Microbial protein (MCP) was indirectly estimated by the following eq. [15]: MCP = (allantoin + uric acid − endogenous PD) × 70 × 6.25/(0.116 × 0.83 × 1000)

**Table 5:** Urine and milk nitrogen (N) excretion in dairy cows fed 3 experimental diets

Items	Treatment^c^			SEM	*P*-value
	AH	L-NFC	H-NFC		
N intake, g/d	504^a^	497^a^	451^b^	1.29	< 0.01
Urinary N excretion, g/d	230^a^	235^a^	191^b^	10.7	0.03
N excretions, % of N intake					
Urinary N	46.2	48.0	42.6	1.79	0.11
Milk N	27.5	26.0	28.0	1.14	0.20

^a-b^Least squares means within a row with different superscripts differ significantly (*P* < 0.05)

^c^AH = Total mixed rations containing alfalfa hay; L-NFC = Total mixed rations containing corn stover with a low content of non-fiber carbohydrates; H-NFC = Total mixed rations containing corn stover formulated to have similar non-fiber carbohydrate concentrations as the AH diet

### Rumen fermentation and plasma variables

The rumen pH at 3 h post-feeding tended to be different (*P* = 0.09) among groups with a lower value for cows fed AH compared to cows fed H-NFC and L-NFC (Table 6). Rumen NH_3_-N concentration tended to be different among 3 groups (*P* = 0.06), with higher concentration for cows fed L-NFC compared to cows fed H-NFC and AH. The concentration of total VFA was higher for cows fed AH compared to cows fed H-NFC (*P* < 0.01) and L-NFC *P* = 0.06) with no difference between cows fed L-NFC and H-NFC (*P* > 0.10). The molar proportion of acetate tended to be different among 3 groups (*P* = 0.06) with lower proportion for cows fed AH compared to cows fed H-NFC and L-NFC, while the propionate proportion was higher for cows fed AH compared to cows fed H-NFC and L-NFC (*P* = 0.02), with a lower ratio of acetate to propionate for cows fed AH compared to cows fed H-NFC (*P* = 0.02). There was no difference (*P* > 0.05) in the molar proportions of butyrate and valerate among the three groups, but the molar proportion of isobutyrate were different among 3 groups (*P* = 0.01).

**Table 6:** Ruminal fermentation variables in dairy cows fed 3 experimental diets

Items^c^	Treatment^d^			SEM	*P*-value
	AH	L-NFC	H-NFC		
pH	6.28	6.42	6.37	0.05	0.09
Ammonia nitrogen, mg/dL	21.4	23.7	21.1	0.85	0.06
Total VFA, mmol/L	120^a^	111^ab^	106^b^	3.08	0.02
Molar proportion, mmol/100 mmol					
Acetate	64.8	65.3	65.8	0.28	0.06
Propionate	20.7^a^	19.7^b^	19.2^b^	0.32	0.02
Butyrate	10.4	10.8	10.6	0.23	0.43
Isobutyrate	0.96^b^	1.03^a^	1.07^a^	0.03	0.01
Valerate	1.40	1.42	1.31	0.04	0.15
Isovalerate	1.72	1.75	1.95	0.07	0.08
Acetate: Propionate	3.15^b^	3.32^ab^	3.44^a^	0.06	0.02

^a-b^Least squares means within a row with different superscripts differ significantly (*P* < 0.05)

^c^The results are from rumen fluid samples collected 3 h post-feeding. VFA = Volatile fatty acids

^d^AH = Total mixed rations containing alfalfa hay; L-NFC = Total mixed rations containing corn stover with a low content of non-fiber carbohydrates; H-NFC = Total mixed rations containing corn stover formulated to have similar non-fiber carbohydrate concentrations as the AH diet

Plasma concentration of total protein tended to be different (*P* = 0.07) among groups with a lower value for cows fed L-NFC compared to cows fed H-NFC and AH (Table 7), while the BUN concentration was highest (*P* < 0.01) for cows fed L-NFC, followed by the AH-fed cows, and the lowest BUN occurred in cows fed H-NFC. Plasma concentrations of albumin (*P* = 0.16), glucose (*P* = 0.21), cholesterol (*P* = 0.30), NEFA (*P* = 0.87), and BHBA (*P* = 0.19) were not different among the three groups.

**Table 7:** Effects of 3 experimental diets on plasma metabolites in cows

Items	Treatment^d^			SEM	*P*-value
	AH	L-NFC	H-NFC		
Total protein, g/L	80.0	78.8	80.4	1.36	0.07
Albumin, g/L	26.5	26.8	26.6	0.40	0.16
Blood urea nitrogen, mmol/L	5.92^b^	6.44^a^	5.61^c^	0.28	< 0.01
Glucose, mmol/L	2.48	2.49	2.44	0.05	0.21
Cholesterol, mmol/L	5.73	5.62	5.67	0.12	0.30
Non-esterified fatty acids, μmol/L	202	204	206	6.81	0.87
β-Hydroxybutyric acid, μmol/L	467	511	470	30.3	0.19

^a-c^Least squares means within a row with different superscripts differ (*P* < 0.05)

^d^AH = Total mixed rations containing alfalfa hay; L-NFC = Total mixed rations containing corn stover with a low content of non-fiber carbohydrates; H-NFC = Total mixed rations containing corn stover formulated to have similar non-fiber carbohydrate concentrations as the AH diet

## Discussion

It is well known that DM intake is closely related to the rumen digesta passage rate, and a rapid passage rate facilitates high DM intake [22–24]. Previous studies regarding the effects of NFC on the passage rate of rumen digesta have been inconsistent. In the study by Linden et al. [25] who supplemented corn stover with corn grain containing high NFC content, the ruminal solid passage rate increased with increasing levels of corn grain, while Batajoo et al. [26] found that rumen digesta passage rate was not affected by the percentage of dietary NFC. There were other reports in which cows consuming diets with lower NFC content had a higher ruminal solid passage rate [27] and liquid passage rate [28]. In the current study, the H-NFC fed cows had a lower DM intake than those fed L-NFC and AH, which might be attributed to the lower ruminal solid passage rate and higher retention time of diet residues in H-NFC fed cows. The chemical composition, physical and digestion characteristics may cause alfalfa particles to have a shorter ruminal retention time compared to other forage particles [29–31], which might result in higher DM intake for cows fed AH compared to cows fed H-NFC even though both diets had a similar NFC content.

Higher DM intake resulted in higher energy intake for the AH-fed cows that produced more milk compared to cows fed H-NFC and L-NFC (Table 3). Milk fat content was lower in AH-fed cows than that in cows fed L-NFC or H-NFC (3.90% vs. 4.25% or 4.11%), indicating that less energy might be needed for per kilogram of milk synthesis in the AH cows, which may also be the reason for more milk produced in AH-fed cows. Although AH-fed cows had lower milk fat content, milk yield was higher in those cows, thus, no significant difference was found in ECM yield among the three groups. Decreased milk fat content resulting from an increased dietary NFC has been reported previously [26, 28], which occurred in part due to changes in ruminal pH and VFA profiles. Increasing NFC content can increase molar proportion of propionate and decrease acetate proportion [26, 32] or have no effect on individual VFA [33]. The results in this study showed no differences in the molar proportion of individual VFA between L-NFC and H-NFC, but a higher propionate proportion was found in the AH-fed cows than in cows fed L-NFC or H-NFC.

The feed cost per kilogram milk was the lowest for cows fed H-NFC compared to other two groups (Table 3), indicating the highest feed efficiency in cows fed H-NFC. In addition, because shifting nitrogen excretion from urine to feces can reduce environmental nitrogen pollution from dairy cows [34], the lower nitrogen excretion from urine in cows fed H-NFC compared with L-NFC and AH (Table 5) indicated that H-NFC is also a better diet for environmental benefit. Overall, from both economic and environmental points of view, the CS contained diet with high NFC content could be a better choice for dairy farms instead of an alfalfa-based diet.

Urinary N [35] and BUN [7] arise largely from excess RDP because ammonia absorbed through the rumen wall may be converted to urea in the liver and excreted in the urine and milk [36]. In our study, the MUN concentrations greater than 18 mg/dL may be attributed to the oversupply of RDP relative to RUP. However, relatively higher rumen ammonia-N content in L-NFC may result in higher urea N in blood, urine and milk, compared to cows fed H-NFC and AH. The proportion of urinary N in N intake also tended to be highest in L-HFC fed cows, suggesting a decreased N conversion compared to cows fed H-NFC and AH. Feed efficiency was also lower in cows fed L-NFC compared to cows fed H-NFC and AH. Urinary concentration of PD and estimated MCP were not different among groups, but urine volume and urinary N excretion in cows fed H-NFC were lower compared to cows fed L-NFC and AH (Table 4). The DM intake is closely related to water intake [37, 38]. The higher urine volume in AH and L-NFC might be attributed to higher DM intake in these two groups.

## Conclusion

Increased NFC content in a diet containing corn stover improved feed efficiency that reached a value similar to the AH diet. The lower concentration of rumen ammonia N as well as lower BUN and MUN and lower urinary N proportion of N ingested in cows fed H-NFC indicated an increased N conversion compared to the cows fed L-NFC.

## Abbreviations

ADF: Acid detergent fiber
AH: Total mixed ration containing alfalfa hay as the main forage
BHBA: β-hydroxybutyric acid
BUN: Blood urea nitrogen
BW: Body weight
CG: Chest girth
CP: Crude protein
CS: Corn stover
DM: Dry matter
H-NFC: Total mixed rations containing corn stover with a high content of non-fiber carbohydrate similar to that of AH
L-NFC: Total mixed rations containing corn stover with a low content of non-fiber carbohydrate
MCP: Microbial protein
ME: Metabolizable energy
MUN: Milk urine nitrogen
N: Nitrogen
NDF: Neutral detergent fiber
NEFA: Non-esterified fatty acids
NFC: Non-fiber carbohydrate
NH_3_-N: Ammonia nitrogen
PD: Purine derivatives
RDP: Rumen degradable protein
RUP: Rumen undegraded protein
SCC: Somatic cell count
TMR: Total mixed ration
TS: Total solids
VFA: Volatile fatty acids
